# The Plasma Mobile, ‘A gift from heaven’: The impact of health technology transfer on trial perceptions and expectations during the Ebola-Tx Trial, Conakry

**DOI:** 10.1371/journal.pntd.0008206

**Published:** 2020-04-22

**Authors:** Almudena Marí Sáez, Maya Ronse, Alexandre Delamou, Nyankoye Haba, Frédéric Bigey, Johan van Griensven, Koen Peeters Grietens

**Affiliations:** 1 Robert Koch Institute, Center for International Health Protection, Berlin, Germany; 2 Institute of Tropical Medicine, Medical Anthropology Unit, Department of Public Health, Antwerp, Belgium; 3 Centre National de Formation et de Recherche en Santé Rurale de Maferinyah, Forécariah, Guinée; 4 Centre d’Excellence Africain pour la prévention et le contrôle des maladies transmissibles (CEA-PCMT), Faculty of health Sciences and Techniques, University Gamal Abdel Nasser of Conakry, Conakry, Guinea; 5 Centre National de Transfusion sanguine, Conakry, Guinea; 6 Établissement Français du Sang Grand-Est, Strasbourg, France; 7 Institute of Tropical Medicine, HIV and Neglected Tropical Diseases Unit, Department of Clinical Sciences, Antwerp, Belgium; Medizinische Universitat Wien, AUSTRIA

## Abstract

During the West African Ebola Virus Disease (EVD) epidemic from 2014 to 2016, a variety of technologies travelled considering the context of the emergency: a highly contagious fast-killing disease outbreak with no known remedy and a rapidly increasing number of cases. The Ebola-Tx clinical trial tested the efficacy of Convalescent Plasma (CP) as a treatment for EVD in Guinea. This paper is based on ethnographic research in the Ebola-Tx trial and focuses on the introduction of a mobile plasma collection centre, referred to as the ‘Plasma Mobile’, equipped with plasmapheresis and pathogen inactivation technologies, as well as how the transfer itself of this technology entailed complex effects on CP donors as trial participants (i.e. providers of the therapeutic product), directly involved staff and more broadly on the trial implementation as a whole. The transfer led to the emergence of a dimension of hope as CP donors hoped that the plasma would cure and, as providers of the therapeutic, hoped it would decrease their stigmatization and the economic impact of the disease. We conclude that, in light of the intricate effects that the transfer of such health technology can entail–in the localization to the specific context, as well as in the consequences they can have on actors involved in the implementation of such technologies–global health technologies should be put at the services of next epidemic and pandemic (preparedness) on condition that they are accompanied by an understanding of the technologies’ own cultural meanings and social understandings.

## Author summary

The Ebola-Tx trial tested the efficacy of Convalescent Plasma (CP) as a treatment for Ebola Virus Disease (EVD) in Guinea during the 2014–2016 West African outbreak. As part of the trial, a bus equipped with plasmapheresis and pathogen inactivation technologies, referred to as the ‘Plasma Mobile’, was used to collect plasma from Ebola survivors, hence recruited as CP donors. Previous studies on clinical trials during the EVD outbreak in West Africa showed positive impact triggered by the introduction of technologies such as improvements in work conditions and care. In our ethnographic research on the Plasma Mobile as transferred technology, we show that technologies contributed to bringing less stress and mistrust in the (highly sensitive issue of the) use of blood products. The organization of the technology in the Plasma Mobile aimed at avoiding mistakes and improving staff performance. In sum, we describe the effects of technologies in staff and CP donors, including the hope for new and improved futures for donors and EVD patients. These results may shed light upon epidemic preparedness, for instance on the need for providing cultural meaning and social understandings to the transfer of technologies.

## Introduction

The West African Ebola Virus Disease (EVD) epidemic in Sierra Leone, Guinea and Liberia (2014–2016) counted 28.000 cases, the highest numbers to date. Guinea registered 3.814 cases and around 1.270 survivors [1]. In comparison, the currently ongoing EVD outbreak in the Democratic Republic of Congo (DRC), in North-Kivu, Ituri and South-Kivu provinces counts 3.310 confirmed cases with 1.168 survivors (on 05.03.2020, [2, 3]. In the initial 2014 international meetings regarding the West African epidemic, the high case fatality rates combined with a lack of proven treatment lead to the WHO recommendation [4] of testing experimental treatments such as the transfusion of convalescent plasma (CP). Special emphasis was put on using clinical trials to generate data to guide the decision-making process. In Guinea, in addition to CP, other drug candidates like Favipiravir [5], ZMapp [6] and Interferon-γ [7] were tested during the epidemic. A recombinant vesicular stomatitis virus–Zaire Ebola virus vaccine (rVSV-ZEBOV) was also evaluated during the epidemic [8] and is currently being used in the ongoing DRC epidemic response.

The Ebola-Tx trial (i.e. *Emergency evaluation of convalescent plasma for Ebola Virus Disease in Guinea*) evaluated the safety and efficacy of CP as potential treatment for EVD [9]. The trial was funded by the European Union and sponsored by the Institute of Tropical Medicine, Antwerp (ITM). A research consortium was created among different research institutions and transfusion services. The use of CP as medicine [10, 11] in the Ebola-Tx trial was proven safe [9] and feasible [12] but did not significantly improve the EVD survival rate [13]. As part of Ebola-Tx, a Plasma Mobile donated by the Bill and Melinda Gates Foundation (BMGF) served as a plasma collection centre. The Plasma Mobile was equipped with plasmapheresis and pathogen inactivation technologies. Plasmapheresis is a medical technology that consists of the extraction of whole blood and subsequent separation of blood components, namely the red cells and the plasma, by decantation or centrifugation. The plasma is selected for therapeutic use and the red cells are returned to the patient or donor. A total of 98 survivors took the initiative to make a CP donation, among which 58 were eligible and tested negative for a transfusion-transmissible infections serological test, meaning their plasma could be used for the trial. About half of those (46.4%) donated more than once. These donations were sufficient to provide the experimental treatment to every patient included in the trial [12].

Aside from Ebola-Tx, Ebola convalescent whole blood (CWB) and CP were also experimented in Sierra Leone and Liberia during the West African epidemic of 2014–2016 [9, 14–16]. The first attempts to use and evaluate CWB took place in Sierra Leona in November 2014 [15]. In Liberia, a clinical trial using CP and a similar Plasma Mobile as the one in Guinea took place in 2014 [3, 14]. In Sierra Leone, the Ebola-CP trial evaluated the use of CP but not in a Plasma Mobile.

In Global Health, biomedical products and processes such as drugs, diagnostics and other technologies are considered universal due to their capacities to ‘travel’ across settings [17] and being simple-to-use [18]. However, the transpositions of innovations and technologies into new settings demand an interaction with local needs [18–21] in order to be operational and respond to expectations created by the inherent foreign cultural perception [22] and symbolic value rather than use value [23]. It is therefore important to document how so-called universal products, local people and contexts interact to make health care innovations ‘work’ or fail [24].

Technology also acts as a cultural device and the transfer of technology can entail cultural meanings related to the disease or the technique [22]. During the EVD epidemic, a variety of types of technologies travelled, ranging from well elaborated ready to use ‘Ebola kits’ [25] to mobile field laboratories [26] home disinfection kits [27], and clinical trials [5, 8, 9, 28–32]. All of these technologies comprise different time-space-limited expectations, such as protection against infections, halting the ongoing epidemic or finding a cure for the EVD.

The technologies of blood transfusion were not new in Guinea Conakry. Blood transfusion has been reported in Guinea since 1955 [33, 34] and has in many African regions been provided mostly by young males, often recruited from places employing an element of coercion like the army, civil servants, students, factory workers and prisoners. These types of blood donors are considered as belonging to the category of (i) unpaid ‘voluntary’ donors, in contrast to (ii) paid donors who received a payment (compensation) for time and effort and (iii) replacement donors (i.e. when a patient’s family is required to donate blood to replace the blood planned for the patient’s transfusion and often leads to finding people willing to be paid in exchange for this service [33, 35]. Blood transfusion, blood donation, and blood sampling have been perceived as part of an extractive colonial medicine, sourced by rumors of stealing of blood [36]. Similar reluctance has been documented from blood donors over time, because of fear of losing strength, the possibility to become infected with other diseases [36, 37] and fears of selling blood [38–41]. In the context of EVD outbreak in Guinea (and West Africa more generally), worries about blood stealing, selling and thus the possibility to transform blood in a commodity with high value were latent [41–45].

The production of plasma was not a new technique at the time of the trial design either but the capacity to produce large amounts of plasma was limited, as it was only being used as part of testing laboratory practices and for rare cases of hemophilic patients. Plasmapheresis was not part of the National Blood Transfusion Centre (*Centre National de Transfusion Sanguine*, hereafter referred to as CNTS) practice and plasma pathogen inactivation processes were not available in the country (as was the case in many others). CWB was thus initially elected as the preferred experimental treatment, in the absence of the appropriate technology (and knowledge on its use) when designing the trial procedures in November 2014.

Our research was embedded in a larger anthropological formative research on the effective and ethical implementation of the Ebola-Tx trial. Technology was central within the trial implementation to produce CP, the final product, and particularly significant in relation to the transfer of the Plasma Mobile and the machines needed to produce this final product. In this manuscript, we thus focus on the following research question: How did the transfer of the new plasmapheresis technologies and the Plasma Mobile impact on the CP donors, the health staff and researchers within the ‘trial communities’ [38] of the Ebola-Tx trial?

We start by introducing the Plasma Mobile with its technology and the role it had in the Ebola-Tx trial as a whole, in addition to the significance that the bus had as a specific space. This space and the organization of it had an impact on the staff working inside it and on the donors. As the Plasma Mobile contained the plasmapheresis machines, we then include a description of the plasma donation process based on our observations. Next, we describe how the technology was also impacted by the context and the steps needed to get the plasmapheresis functioning in Conakry, i.e. the transferring and localization of the technology to the context. Finally, we show how the technology (the Plasma Mobile and the plasmapheresis-related technologies) and the production of CP had effects on the trial communities, from distinction between blood and plasma, to a better work performance of the staff working at the CNTS, to finally the generation of hope.

## Methods

### Data collection

Qualitative ethnographic methods were used by the anthropologists for data collection. Participant observation, including informal conversations, was conducted in the Plasma Mobile, during the plasma donation process, and during daily interactions with trial communities. The Ebola-Tx trial communities included the CNTS staff, *Médecins Sans Frontières* (MSF) staff, CP donors, EVD survivors (and the Survivors Association) more broadly, expatriate staff (from ITM, MSF, the French Blood Centre (*Etablissement Français du Sang*, hereafter referred to as EFS) and by extension other stakeholders and groups affected by the trial. Field notes were written on a daily basis. In addition, a total of 23 individual semi-structured interviews and 22 focus group discussions were conducted with CNTS staff working on the plasma collection process, MSF teams carrying out the CP transfusion to EVD patients, ITM and EFS staff, relatives of EVD patients, EVD survivors and CP donors between January 2015 and January 2018.

### Sampling

Informants were selected based on theoretical sampling, which involved the purposive selection of participants based on emergent results from fieldwork. The criteria for participants’ selection included multiple levels of involvement in the Ebola-Tx trial (staff involved in the trial, EVD survivors in contact with trial team, CP donors), variation in gender, age, socio-economic status and ethnicity/nationality.

### Data analysis

Categories and relevant patterns were identified based on the intermediate analysis of emerging data during the implementation of the trial process. Emerging findings were constantly cross-checked during additional interviews. The recurrent themes were analysed and categorised from the interviews. Retroductive analysis combined continuously collected data with literature on mobile technologies, blood sampling and clinical trials and theory from related medical anthropological literature, Science and Technology Studies theory, apheresis and EVD literature.

### Ethical approval

Oral informed consent was obtained from all adult participants. Oral rather than written consent was preferred in this study due to the sensitive nature of the study, including (i) the high illiteracy in the study population challenging the practical obtaining of written consent; (ii) written consent might seem a breach of confidentiality by the participants; (iii) written consent, requiring a signature and therefore introducing formality in the procedure, affects trust between respondents and researchers and consequently willingness to participate in the study, but also introduces bias which reduces data quality. Informed consent was documented in writing by the person who obtained the consent and, if present, by a witness. Ethical approval for this qualitative study, including the use of oral consent, was obtained from the National Ethics Committee for Health Research in Guinea (131/CNERS/16), MSF’s Ethical Review Board, and the ITM’s Institutional Review Board (IRB/AB/ac?092 ref:1098/16).

## Results

### The integration of the Plasma Mobile in the Ebola-Tx trial

#### The Ebola-Tx trial partners

In Conakry, the implementation of the Ebola-Tx trial consisted of the collaboration between (i) ITM’s research team and (ii) MSF with, as partners on the ground, (i) the CNTS; (ii) the Maferinyah National Centre for Training and Research in addition to (iii) EFS and the (iv) National Ebola Coordination Cell [9, 12]. Cf. the Methods section for acronyms.

The CNTS was the national institution in charge of the collection, laboratory analysis, storage and distribution of WB in Conakry. The CNTS was also responsible for the coordination of other blood transfusion centres in the capital’s hospitals and several regional blood transfusion centres (in Kindia, Labé, Kankan and N’Zérékoré).

#### The introduction of the Plasma Mobile

**Fig 1:**
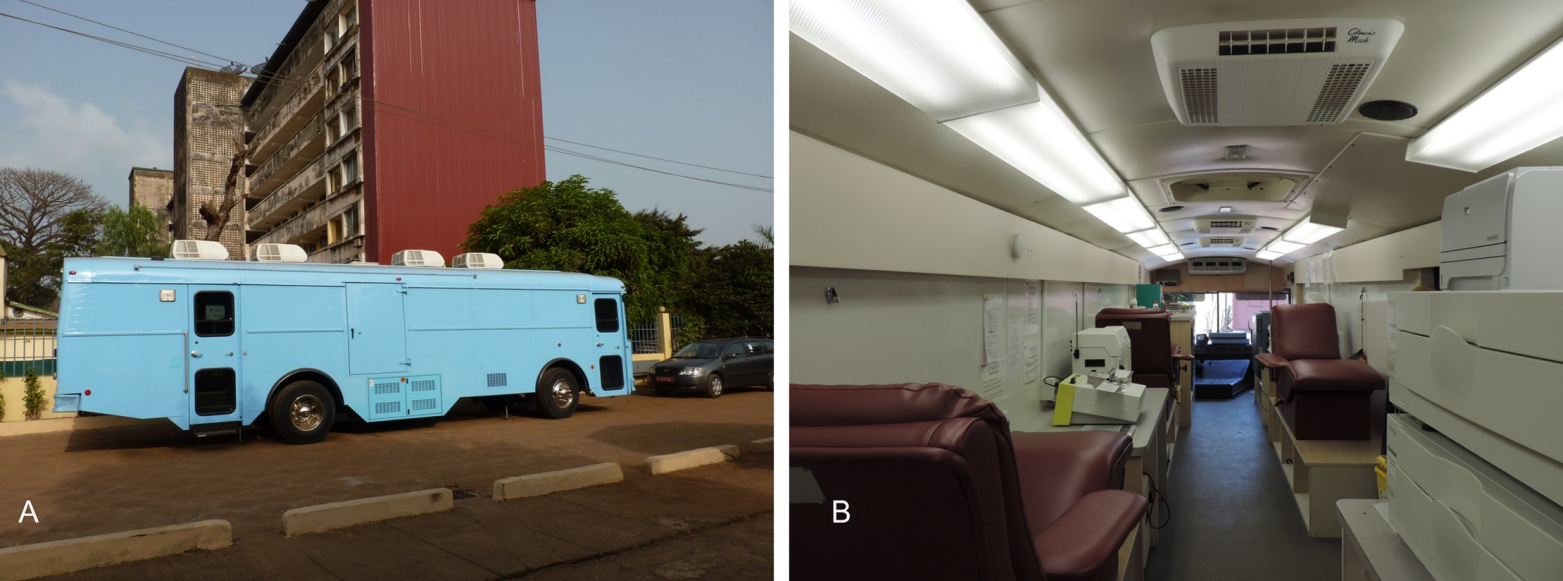
‘Le bus’. The exterior (a) and interior (b) view.

At the end of 2014, unexpectedly the BMGF donated a fully equipped plasmapheresis unit, located in a bus, to the Ministry of Health in Guinea. The vehicle was donated to and parked at the CNTS in Conakry (Fig 1A). It later became known in English as the Plasma Mobile, and commonly referred to in French as ‘*le* bus’. One key stakeholder described the arrival as follows: “*The bus arrived against all odds*. *[…] My first reaction was*: *these people are coming from heaven*”. The introduction of the transferred technology allowed the Ebola-Tx trial to switch from CWB to CP. The trial was originally designed to use CWB but ended up using CP. Compared to other experimental treatments tested during the EVD epidemic, CWB and CP had the advantage of being locally available, in addition to the antibodies in the CWB or CP being active against the specific virus strain in circulation [46]. CP, instead of CWB, then became the preferable blood product to use as potential treatment for EVD for several reasons, including the fact that (i) plasma donors are supposed to recover quickly and can repetitively donate every two weeks as opposed to whole blood for which donors generally tend to feel weaker post-donation with longer estimated recovery time (a maximum of four donations per year per donor is allowed according to the national blood transfusion policy); (ii) plasma entails less risk of blood group incompatibility as there is no rhesus factor. Furthermore, (iii) pathogen reduction technologies can be applied to plasma, which was a consistent safety advantage in the epidemiological context of Guinea.

#### The construction and use of space in the Plasma Mobile

The Plasma Mobile contained a small room to perform the clinical interview and donor counselling, three plasmapheresis Haemonetics machines, four donor sofas, two refrigerators, a weighting scale, a sterile connection device and two illuminators for Amotosalen pathogen inactivation (INTERCEPT^TM^ Blood System, Cerus) (Fig 1B). The Plasma Mobile was made to function with American electrical grid, language, parameters and calibration standards. The Plasma Mobile was considered the preferable location for CP donation and pathogen inactivation for hygiene purposes, as well as its stability and independence from the often interrupted main electric system that was not compatible with US electrical standards.

EFS’s teams had reflected on the way to organize the space inside the Plasma Mobile during trial preparations in order to optimize it, avoid mistakes from staff and contamination as well as to provide better care to donors. Consequently, plasmapheresis machines and the donor ‘cycle’ were organized following a flow diagram: donor entry and exit from different doors, separation in time of plasma donation and preparation phases, restriction of CP labelling to a specific place as it was perceived to be a critical step. The plasmapheresis and the pathogen inactivation practices also demanded high asepsis; hence the bus was a better controlled space (compared to the CNTS building).

The Plasma Mobile (see Fig 1) represented an exclusive and restricted space for health staff. The closed, access-controlled bus, and the specific appointed tasks, helped the staff to accomplish their role properly, allowing them to concentrate and avoid mistakes in following the protocols, in setting-up the machines and in their interactions with the donor. The local staff working in the Plasma Mobile also perceived the space as beneficial for concentration and focus on the CP donation: “*I like the bus*. *It is better here*, *though it is a tiny space*. *This work*, *you can’t do it if you are talking with someone*. *With the plasmapheresis you can’t do two things at the same time*. *You need to be focused*, *you can’t make a mistake*” (CNTS, IDI, Conakry, 05.03.2015).

In addition, staff working in plasmapheresis knew how Ebola survivors were stigmatized in their neighborhoods as “*people run away from them*” (CNTS, IDI, Conakry, 02.03.2015)–and they made an effort to make the donors feel welcome when they came for a plasma donation. In that sense, the Plasma Mobile allowed for a certain intimacy and social proximity between staff and donors that more open and public spaces would not have allowed.

For donors however, the Plasma Mobile as isolated space was interpreted with ambiguity. For some it was a space adapted to the needs of CP donors, peaceful and secured. Others, conversely, feared entering an unknown restricted space, not knowing what and who to expect inside, and demanding increased trust in health staff. This fear was described by several donors to have disappeared by the reassuring presence of witnesses, e.g. acquaintances, other donors or ‘white’ staff. At the same time, some participants felt reassured by the location of the Plasma Mobile within the CNTS and within the larger Donka Hospital complex. Donors had been treated at the Ebola Treatment Unit (ETU) set-up by MSF in this same hospital and was therefore perceived by some donors to be a public place of care, where other people (‘witnesses’) would always be present, and as such constituted a trustworthy space. *“A3*: *I will say*, *where they treated me*, *if it’s not there that the bus is located*, *somewhere else outside of Donka*, *I cannot go there*. *When I go there*, *they will go to kill me*. *[…] Q2*: *You too*? *[…] [I]f it was someplace else*, *would you go*? *A2*: *If it was somewhere else*, *I couldn’t go because at Donka*, *it’s crowded*. *It’s a public place where they treat sick people*. *And what they’re going to do to you is in front of witnesses*.*” (EVD survivors*, *FGD*, *13*.*06*.*2015)*.

#### The Plasmapheresis Process

**Fig 2:**
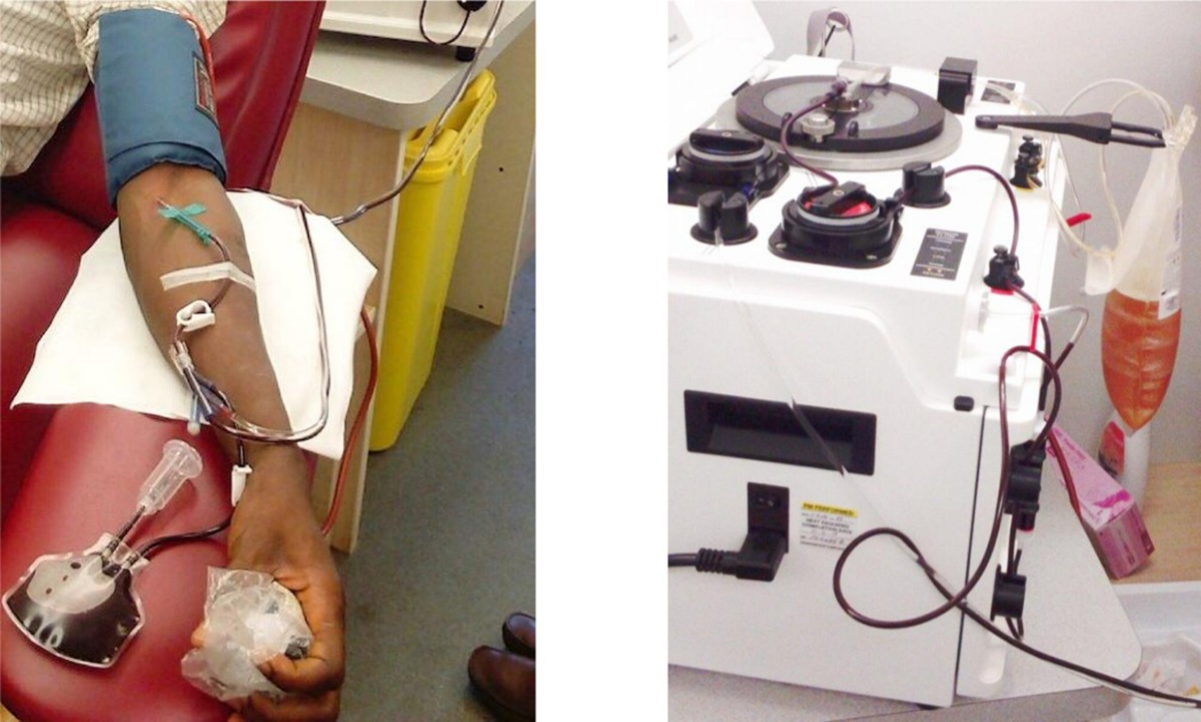
Plasmapheresis. Plasmapheresis during CP donation.

On a donation day, a focal point from the Survivors Association (*Association des Personnes Guéries et Affectées d’Ebola en Guinée*) would briefly welcome CP donors into a specific room dedicated to this purpose inside the CNTS building, and accompany her/him to the Plasma Mobile. Subsequently a doctor would conduct the informed consent procedure and examine the donor. After consenting and being declared fit for donation, the donor would be appointed to one of the donor chairs. A single use plasmapheresis kit would then be connected into (plasm)apheresis machine, and a needle inserted into the donor’s arm to link her/his vein to the machine. The CWB would first flow into 4 blood tubes of 7 ml, and subsequently flow into the apheresis device while being anticoagulated with citrate (Fig 2). After a collection of CWB, the machine would centrifuge the CWB, separating plasma from the red blood cells. The plasma would consequently be extracted and collected in a bag, and the red blood cells returned to the donor. This apheresis cycle would be repeated 3 to 5 times, depending on the hydration level/concentration of red blood cells, until a maximum of 875 ml of CP (including citrate) would be obtained. The whole procedure required approximately one hour.

### Transferring technology

#### Initial planned setting of the technology transfer

Before the start of the trial, experts from the ITM and the EFS, in communication with Clinical RM, assured the set-up and adaptation of the needed technologies inside and outside the Plasma Mobile. EFS trained the local staff in charge of the plasmapheresis: a doctor to conduct the preliminary medical interview, a technician to be in charge of connecting the donor to the apheresis machine and a nurse overseeing the donors during the donation [12]. Additional health staff was trained for the pathogen inactivation, preparation and qualification of the CP for use by laboratory experts from ITM and EFS. The qualification of the CP and the storage was carried out inside the CNTS building.

#### Language

Initially, the language display in English on the plasmapheresis machines couldn’t be changed. Consequently, the EFS team had to teach local staff, who had no English proficiency, to mechanically follow each step shown on the screen of the machine. At the same time, a pictorial guide was created for the process and displayed on top of the machine. According to some of the staff, this was the moment when the relationship with the machine was the most difficult: “*[The machine] was in English*, *and I do not understand English*. *We started learning how to practice before interpreting the message*” (CNTS, IDI, Conakry, 05.03.2015).

#### Metrics

The EFS, in line with the national and other trial partners’ standards, decided to work with the International System of Units/Metric values (kg, cm, ml) instead of the Imperial System (pounds, inches, ounces). The scales used to weight the plasma units in the bus were in ounces while the ones in the preparation lab (in the CNTS) were in grams, the latter helped the staff to confirm the correct weight.

### Localizing the transferred technology: challenges, successes and failures of an adaptation

#### Turning an American machine into a Guinean one

A month after its initial use, a French technician from the manufacturers (Haemonetics) went to Conakry to change the language, values and ratios of the software into French. The final choice was seen as “*changing the American machine into a French machine*” (EFS, IDI, Conakry, 13.02.2015), attempting at coming closer to the local context and facilitating the appropriation by the local staff.

#### Kits

As a consequence of the shift to French parameters, the plasmapheresis machines required specific disposable kits. These entailed to use another anticoagulant to get the appropriate ratio by volume of blood to be processed. A new delivery of kits of the Haemonetics apheresis device was shipped from France to the CNTS. The new apheresis kits had the particularity of being ‘secured’ because they were pre-assembled thus ready-to-use and had a specific connection system to reduce potential mistakes from staff. The American kits, in contrast, needed to be assembled before use.

#### Power system

The conversion of American standards of power values to the Guinean system was needed as the American machines were ready to be used on 110 volts/60Hz instead of 220 volts/50Hz. Due to the complexity of the patchwork status of the electric circuit and supply, it became more efficient–after several attempts to adapt the system between the CNTS building and the Plasma Mobile–to get electricity using the Plasma Mobile’s own power generator rather than connecting it to the main power supply. In addition, a separate power generator was purchased to assure the procedures that were needed inside the CNTS building, including the qualification, laboratory testing and storage of blood products.

#### Timeliness of the technology localization process

The process was seen by staff as achieving the change from a US to a French system. In general, the understanding of the procedure took time and the relationship with the technology was perceived as something complex and distant rather than being an intuitive and accelerated tool. Rather than expediting the trial set-up, the technology initially delayed the process at certain levels. The transfer of the technology was superficial, time-consuming and not adapted to the local reality, hampering ownership by local staff during and after the trial. The sustainability of the new technology was also anticipated as “unlikely to be an optimal choice”[46] for the National blood services. The cost of running the bus post-trial for a plasmapheresis would be high for an already underfunded blood bank service.

From the donors’ perspective, this process of localization per se was not experienced directly as they did not play an active role and were only involved in the final part of the localization process.

### Effects of the technology transfer on staff and donors

#### Blood vs. plasma and donation concerns

The technology helped, in some cases, to have a comprehensive idea of the difference between blood and plasma, “*the technology allowed people to understand that we were not emptying them of their blood*, *but of another liquid*, *and that we were replacing it with another liquid*, *their own red cells*” (CNTS, IDI, Conakry, 27.01.2017). Donors acknowledged that the liquid circulating in the tubes from the arm to the machine was blood as it was red, and were able to distinguish it from the yellow plasma in the (plasma) collection bag at the end of the donation. “*We didn’t know the difference between blood and plasma*. *[…] With the plasmapheresis*, *a liquid part of the blood was removed*, *the plasma*. *[…] The technology helps to understand not only the separation*, *but also the mechanism*. *Otherwise we would have asked ourselves the question*: *‘how will the separation be done*?*’ But just through the sampling [i*.*e*. *the plasmapheresis process] already*, *the packaging is there*, *there is the plasma”*. (EVD survivors, FGD, Conakry, 26.01.2017).

Donors had different expectations and experiences during the donation process [11]. Donors sometimes voiced a certain distress related to the Plasma Mobile, to the machine per se, to the unknown experience the plasmapheresis entailed, to the needles and to the pain of the process as well as the post-donation consequences (dizziness, weakness, falling ill…). *"They gave me food*. *After this*, *when I was going*, *before entering the bus*, *I was really scared*. *(…) Not only was I scared of the machines*, *but also of what they told me about giving blood*. *It was all the blood we were giving*. *I was scared*. *(…) Now when I entered (…) it didn’t even take one hour of time*, *I was already done with donating”* (EVD survivor, FGD, Coyah, 13.06.2015). For some, once they were inside the bus and the process started, they felt obliged to continue, as one informant voiced: “*Inside [the bus]*, *when you see the machine you are scared*. *But then*, *everything you want to do*, *you need to make up your mind before*” (EVD survivor, IDI, Conakry, 14.03.2015). For others, the time during the donation was spent talking with the staff, looking at their phones or relaxing, and was very distressful. “*I took courage*. *They [staff] took one of my veins and started*. *I didn’t feel dizzy or anything*. *[…] To complete the process I did one hour* (EVD survivor, IDI, Conakry, 14.03.2015).

During the early trial stages, in the context of a climate of suspicion that was generally prevailing, some survivors and local staff were confused about the intentions of the trial using CP and they feared that the plasma would be used to infect people or for lucrative ‘business’ purposes by earning money out of medicines made from EVD survivors’ plasma. Throughout the study, concerns about the ‘blood’, the quantity extracted and its purpose remained a worry for donors.

#### Specific and targeted care for survivors

To create a relaxing atmosphere and make this exceptional situation seem more normal and routine, staff would turn on the bus radio at times. In addition, the available air-conditioning was considered as a factor of comfort while working or donating plasma, contributing to the meaning of being in a cutting-edge place, as air-conditioning is associated with high-class standards.

On top of the close relationship between some staff and donors, some donors felt they received full and personalized attention as the machines were regulated to collect the adapted amount of plasma calculated for each donor based on biological factors. The donation time was different for each survivor. Once connected to the machine the donor could only be *detached* at the end of the plasmapheresis. The high amount of liquids consumed to boost hydration levels made some donors feel the urge to urinate during the plasma extraction process. For male donors, the staff adapted to this by offering them a half-cut plastic bottle to urinate in while remaining connected to the machine, which was considered a relief. Female donors couldn’t benefit from this solution. The separate space that the Plasma Mobile constituted (from the CNTS and other hospital buildings in the neighbourhood), also contributed to the feeling of targeted care in the sense of confidentiality, where their specific status as EVD survivors and situation could be discussed, out of sight from more public attention.

#### Empowered staff

The bus and the newly introduced technologies were perceived to increase the performance of local professional practice. Staff conducting the plasmapheresis felt more professional as they were trained in the manipulation of the new equipment as well as in following strict standard operating procedures, regularly filling in monitoring forms (of patients and equipment), and improving hygiene practices, all of which were perceived to lead to rigorous work standards. This contributed to staff’s confidence, and consequently, their motivation.

The technology, as part of the trial in general, also brought better work conditions. Among others factors, in addition to taking part in the response to control the EVD epidemic, these conditions included being part of a scientific research team working closely with international experts, incentives for local staff, and getting access to training and Hi-Tec. The local introduction of the technology was considered an opportunity: “*we didn’t go and learn anywhere else*, *we have learned here*, *in this place*. *The work tools have come and found us here*” (CNTS, FGD, Conakry, 22.06.2015).

Staff felt that all conditions (material, technological, educational and financial) were present to carry out a committed and dedicated job, which consequently also led to an improved relationship with CP donors.

#### The generation of hope

Plasma is a therapeutic which needs the intervention of technologies to exist on its own, i.e. to get separated from the blood it constitutes, but also in order to get treated before being safely transfused to patients. When people taking part in this research talked about the therapeutic product, plasma, they referred most of the time to the final product: the ‘blood’ that can cure; i.e. the plasma can give more opportunities of survival. A main consequence of those projections for the plasma as a final therapeutic product was the production of hope in the cure and through the technology that was embedded in the process to obtain the plasma. Plasmapheresis technology (and to a larger extend the Plasma Mobile) was inexistent in Guinea before this trial. As a result, the introduction of this technology, which directly involved the EVD survivors in the potential cure of EVD, triggered the emergence of hope. CP donors in the trial talked about plasma as the possibility to cure other people, and for CP donors this justified their aim to undergo the plasmapheresis. EVD survivors who had lost many family members, neighbours and friends, sometimes in front of their eyes, expressed that these lost relatives could have still been there that day if they had benefited from a cure. One donor explained her point of view about the trial. When she was infected with EVD, she was admitted with her brother in the same ETU: “*I have never given blood*. *And this [plasma donation] I told myself*, *now is the time*. *This is also for saving people’s lives*. *And this I know about*, *I’ve been through this*. *I have seen it [the Ebola disease]*, *I know what it means*. *[…] It [CP] could have saved him [my brother]*, *of that I am sure*. *[*…*] You know*, *[…] he wasn’t eating [anymore]*. *If he would have received it very early*, *and if the plasma would have arrived here very early*, *this would have helped him a lot*.” (EVD Survivor, IDI, Conakry 13.03.2015).

Some CP donors were, under particular circumstances, combining several roles within the trial: e.g. being a survivor, a CP donor and working at the ETU, which allowed them to follow the use of CP and reaffirm their decisions about CP donation: *“yes*, *other survivors asked me if I gave [plasma]*, *and I said*: *‘yes*, *I gave’*. *They told me*: *‘ah you have given without thinking*, *eh*?*’ I said*: *‘yes*, *I gave as it is to save lives*. *And I have seen what they [trial staff] are doing inside*. *They are giving [to EVD patients] what we gave [the plasma]*.’” (EVD Survivor, IDI, Conakry 13.03.2015).

CP represented that possibility of a cure. Health care providers working at the ETU also talked about the CP as the potential solution after a year into the EVD epidemic. One of the medical doctors working at the ETU explained: “*We have started something [a clinical trial] which opens different ways*, *different doors and research points*. *Q*: *And for you this was something that needed to be done*? *R*: *Yes*, *absolutely*. *[…] People [other team members working at the ETU] were convinced this was something that needed to be done*. *It’s just that at the beginning*, *people thought it was the ‘magic bullet’*. *[…] That needed ‘tac’*, *to be given*, *and ‘tac’*, *you have a result*. *And it’s not like this*. *Anyways*, *the hope linked to the trial is high*.” (Staff ETU, IDI, Conakry 03.03.2015).

The plasma and the technologies to obtain it thus acted as a catalyst for hope: hope for researchers to prove plasma as being safe and effective against EVD–“*It was the moment*. *We waited too long*. *[…] Plasma is a good hypothesis and it has been proven for other diseases*. *And also to understand what are Ebola antibodies*, *the different types*, *and whether they help*. *[…] Also for the vaccine*.” (ITM staff, IDI, Conakry, 24.02.2015)–; hope for local healthcare workers to contribute to finding a treatment that would cure their patients, alleviate their work and convey this hope to these patients; as well as hope for EVD survivors to embody the solution for Ebola in their country–”*We are doing this out of patriotism*. *To help the Nation*. *It is like this*, *that’s all*. *Otherwise no one can pay money for donating plasma*. *That’s not possible*” (EVD survivor, IDI, 23.02.2015).

Survivors also hereby hoped to gain social recognition and personal self-esteem as well as reintegration in society: *“This is a clinical trial*, *we will see if our blood*, *our plasma can really give a chance of survival to someone who has Ebola*. *[…] I have the courage to give my blood again*, *my plasma*, *to treat someone with Ebola*. *If the person manages to survive with my plasma*, *I will be proud*. *[…] Then*, *even the people who are stigmatizing us in the neighbourhoods will say*: *‘really*, *these people [EVD survivors] are cured*’”(EVD Survivor, IDI, Conakry 14.03.2015). The hope that CP, the experimental treatment, would be effective and thereby re-strengthen survivors’ identities, made survivors decide to accept the transferred technology as part of the general Ebola-Tx trial by becoming CP donors.

## Discussion

We conducted a social study on the transfer and process of localizing technology and questioned which impact these had on people participating in the Ebola-Tx trial, from staff working on the set-up of the Plasma Mobile and the plasmapheresis process, to the donors and staff transfusing the plasma.

### The localization of the transferred technology

Our article illustrates how the technologies introduced in an emergency clinical trial like the Ebola-Tx trial in Conakry and their impact need to be studied, and some universal ideas about global technologies questioned [18]. Assumptions that technology will accelerate the starting of a trial, or that technologies are mobile and easily adaptable and hence by transferring the related standardized knowledge and skills to the local staff they will smoothly accommodate to the machinery, are examples of such assumptions that need to be questioned. This paper presents evidence on the need to adapt the plasmapheresis machine to work in a different context, such as installing different software, facilitating its use by non-English speakers. The plasmapheresis with French parameters also impacted the use of new kits, additionally reducing possible mistakes from the staff. An illustration of how context can make same technologies work differently is the Liberia (Monrovia) case where the same Plasma Mobile was transferred and was reported to be technically quite easy to implement [14]. Our study in Conakry proved that the technology needed a completely different adaptation to the national system and metrics leading to a deceleration of the trial. Intensive training of and adapted to local staff was essential to allow for their appropriation and ownership and alternative solutions needed to be found for incompatibilities in electrical power and languages systems between the local context and the technology’s origin. But the adaptation didn’t permit the use of the Plasma Mobile beyond its use in the trial. It has been parked without being used at the CNTS since the closure of the trial. There are now plans to start using the bus, without the plasmapheresis machines, for outreach (whole) blood collection campaigns. The low level of request for plasma treatment and the lack of financial sustainability makes the plasmapheresis machines more challenging to use in the near future.

Technology has impacts on the understanding and value of the blood products. R. Lynch and S. Cohn studied in the UK blood service centers how blood acquired value through all the transformations [47]. For plasma, our study showed that the technology transforming body fluid into the final product was also important because it helped to illuminate the difference between blood and plasma. Transparency in the plasma machine process of separating blood components and returning red cells to the donor during the Ebola-Tx trial, reduced the stress about the final use of plasma for some donors.

### Social construction and use of space

The social construction and use of space in the Plasma Mobile showed largely positive impact on the CNTS staff’s work performance as well the privacy some survivors needed to feel comfortable during the donation and get the feeling of receiving dedicated care. Studies about organization of, use of and relationships in space have long instructed us about the importance of space and the social practice embedded in it [48]. Jessica Mesman in her study on the organization of work in a neonatal intensive care unit [49] showed how the ordering of people and actions in space aimed at avoiding mistakes, making patient safety a spatial accomplishment [49]. The Plasma Mobile and the plasmapheresis followed a similar logic. The organization of the plasmapheresis and pathogen reduction machines and the procedures to manipulate them in the bus followed a scheme to make the use of the technology in its designated space more coherent and safer, aiming to avoid mistakes and contamination. In the same line, the flow of donors and activities were organized to improve performance. People working inside the bus embodied this logic and consequently felt more performant in the Plasma Mobile. At the same time, both CNTS staff and some survivors perceived that being in a separate space like the bus was beneficial to keep the donations confidential, tailored to the survivors’ needs and to avoid being criticized or misunderstood by their relatives, which would have a negative impact on their social life as explained in previous work [11] notwithstanding other survivors considered such a closed and restricted space suspicious. This restricted space was, however, part of the larger space of the Donka Hospital that was considered public and open, helping donors to accept and trust the more restricted space of the Plasma Mobile.

### The localization of hope

In Global Health interventions, hope is inscribed in a specific time-space, in objects and products [50]. Hope in biomedicine has been studied from the point of view of patients as receptors of ‘last hope’ treatment, therapies or technologies in terms of risks and benefits (examples in [50, 51]. Egg freezing and cord blood stem cell banks have also been seen as ‘regimes of hope’ for future gestations and therapies [52, 53].

This study had as particularity that the involvement of EVD survivors as donors triggered a type of hope that is different from the hope of patients undergoing an experimental treatment for their personal health. The Ebola-Tx trial allowed us to explore what effects the CP as potential therapies and cutting-edge technologies had on the people providing the potential treatment, what risks and benefits these would have for them beyond the material reimbursement of their time and transportation.

CP represented a potential cure. In order to acquire it, the CP donor needed the plasmapheresis technologies. By this mean the plasmapheresis machines, the pathogen reduction machines and the Plasma Mobile more broadly transformed her/his body fluid into a therapy, as plasma is thought to be “a product which is a finished pharmaceutical” [54]. Time–space hopes were inscribed in CP donors’ past events, the EVD infection and the time spent at the ETU. They connected this specific past experience with the future possibility of their plasma curing the patients admitted in the ETUs. Donors wanted to prevent other people admitted in the ETU from experiencing suffering, fear, stress and loss of relatives. Additionally, in this case, the survivors’ hopes transcended the cure of fellow EVD patients and implied their own recognition in society by showing that their ‘survivor’ status allowed to cure other people. As described in Sierra Leone [55], some Ebola survivors faced challenges reintegrating in their communities during the EVD outbreak despite being presented as ‘Ebola heroes’ in public campaigns with posters displayed in survivors clinics and public spaces in Freetown and other districts. Ebola survivors in Sierra Leone were also thought to be a potential source of contamination, by the presence of Ebola virus in their semen [56, 57]. Guinean Ebola survivors faced similar discrimination from fear of contagion even before the general public knew about the persistence of Ebola virus particles in semen [58]. CP donation created hope in the potential for Ebola survivors to stop being regarded as ‘bodies of risk’, by proving that they were cured and able to cure other people. In this sense hope helped to imagine new futures for donors and EVD patients and created ‘intangible motivations’ that fashioned individual decision-making in taking part in the trial [45].

One of the clinical trials that took place in the same period of time as Ebola-Tx in Guinea, was the ‘Jiki trial’. Jiki means ‘hope’ in Mandingo language [59]. This hope was not an exclusive experience and motivation for EVD patients’ achievements of survival. Le Marcis explained how the hope patients had in the new drug introduced a change in the medical staff performances and how the medical staff related with patients [60]. In our study, medical and research staff also developed different forms of hope.

These different forms of hope, of survivors, researchers and healthcare workers were intertwined yet distinct in certain aspects. The different positions, experiences within the trial hence understandings thereof shaped the hope(s) of the different members of these trial communities, converging in the common/shared hope for a solution to this epidemic, but distinguishing them in the individual hopes they triggered such as hope for reintegration in society versus hope in advancing scientific knowledge on the virus and therapy, versus hope for a better outcome and facilitated care of patients. Both distinct and common forms of hopes united different members of the trial communities towards the common goal of the trial.

## Conclusion

Our results showed how, as in other clinical trials during the EVD outbreak in West Africa, technologies brought some expectations for better work conditions [45, 61], better work performance [60], better care provided to the patients [45] as well as less stress and mistrust about the use of blood products.

It is therefore also important to analyse the context in which hope ‘assumes meaning and is mobilized’, as Petersen and Seer did [62]. In line with Carlos Novas [63] we think that the political economy of hope acts as an impulse for people’s aspirations beyond the immediate effect of the technology. Conversely to the study of Novas, we did not study the hopes and social practices of patients but of those who provided the therapeutic, staff and researchers. All wanted to contribute to the response for the EVD outbreak; they wanted to contribute to research development and find a cure for EVD.

Technologies are increasingly being put at the service of global health interventions. We have shown how technologies enfolded in peoples’ actions, expectations and hopes in the time-space of an emergency EVD clinical trial using convalescent plasma. In light of the intricate effects that the transfer of such health technology can entail–either in the localization to the specific context as well as in the impacts they can have on actors involved in the implementation of such technologies–, our analysis suggests that global health technologies should indeed be put at the services of the next epidemic and pandemic (preparedness) on condition that they get accompanied with the technologies’ own cultural meanings and social understandings of the space and context that make them work.

### Field challenges and limitations

Working in the context of an EVD epidemic had consequences for our research: we faced time constraints in the design, the collection and analysis of the data at the same time as we were providing real-time feed-back to the trial’s scientist and implementers as well as providing practical help amidst the emergency. This multitask role required to conduct a reflexive exercise on our work as anthropologist and identify opportunities and challenges of being in between.
